# Validity of the Global Leadership Initiative on Malnutrition criteria in East Asian patients with gastric cancer: a comprehensive narrative review

**DOI:** 10.3389/fnut.2024.1462487

**Published:** 2024-11-20

**Authors:** Jian Wang, Bingyue Liu, Jianxin Chen

**Affiliations:** ^1^The Third Clinical Medical College of Zhejiang Chinese Medical University, Hangzhou, Zhejiang, China; ^2^Hangzhou Zhanshi Traditional Chinese Hospital of Orthopaedics, Hangzhou, Zhejiang, China; ^3^Department of Medical Oncology, The Quzhou Affiliated Hospital of Wenzhou Medical University, Quzhou People's Hospital, Quzhou, Zhejiang, China

**Keywords:** GLIM, gastric cancer, diagnosis, prognosis, postoperative complications

## Abstract

**Background:**

Malnutrition is a significant public health issue for patients with gastric cancer, particularly in East Asia, the region most affected globally. In response to the absence of adequate tools for assessing nutritional status, the Global Leadership Initiative on Malnutrition (GLIM) criteria were established in 2018, aiming to standardize the diagnosis of malnutrition. However, there is no consensus on the value of GLIM criteria for evaluating the nutritional status of patients with gastric cancer in East Asia. Given these facts, our study aimed to assess the validity of the GLIM criteria in East Asian patients with gastric cancer.

**Methods:**

We conducted a rapid critical review of available literature, summarizing the existing problems in GLIM applications and possible improvement directions. After systematically summarizing the literature published in PubMed, Web of Science, and Cochrane Library, a total of 13 articles involving 7,679 cases were included in this study.

**Results:**

The results indicated a lack of sufficient data on sensitivity and specificity to fully validate the GLIM criteria for diagnosing malnutrition in East Asian patients with gastric cancer. Additionally, some studies have reported moderate agreement between the GLIM and the PG-SGA. Furthermore, malnutrition defined by GLIM is a risk factor for short and long-term outcomes in East Asian patients with gastric cancer. However, the prognostic effect of moderate malnutrition on these patients remains controversial.

**Conclusion:**

Despite being in the early application stages, GLIM has shown promising potential in diagnosing and predicting the prognosis of malnutrition. However, future research should incorporate more comprehensive validity parameters, including sensitivity, specificity, and PPV/NPV, to achieve a more thorough understanding of GLIM’s diagnostic efficacy. Furthermore, further optimization of GLIM is necessary to address the needs of more diverse populations and situations.

## Highlights

- Malnutrition defined by GLIM is a risk factor for long-term prognosis and postoperative complications in East Asian gastric cancer patients.
- The impact of moderate malnutrition defined by GLIM on the long-term prognosis of gastric cancer remains controversial.
- GLIM has a strong diagnostic value for diagnosing malnutrition in East Asian gastric cancer patients.

## 1 Introduction

Gastric cancer is a global health concern characterized by high aggressiveness and poor prognosis (1). Although the incidence and mortality rates of gastric cancer have recently declined, it remains the fifth most common malignancy worldwide and the fourth most lethal malignancy, accounting for 8.2% of all cancer deaths (2–4). The 5-year survival rate is only 20% (5). The incidence and mortality rates of gastric cancer exhibit significant variation across different regions globally (3). Notably, the age-standardized incidence rate (ASIR; 14.3/100,000) and age-standardized mortality rate (ASMR; 10.0/100,000) of Asian gastric cancer are the highest globally, with South Korea, Japan, and China being the most affected regions (6). Additionally, East Asian patients with gastric cancer had higher ASIR and ASMR rates of 22.4/100,000 and 14.6/100,000, respectively (7). Furthermore, nutritional disorders are present in over half of patients with gastric cancer, significantly impacting their survival and quality of life. This poor nutritional status arises not only from gastric cancer itself, which leads to symptoms such as loss of appetite, nausea, vomiting, and abdominal pain, but is also influenced by chemotherapy agents and post-gastrectomy syndromes (8). Given these challenges, it is necessary to improve the management of gastric cancer from many aspects, including risk factors, early screening, treatment, diagnosis, and prognosis.

Malnutrition is a state of imbalance in body composition and impairment of physical and mental functions caused by abnormal intake or absorption of nutrients (9, 10). It is an independent risk factor affecting prognosis and exists in all stages of gastric cancer (11, 12). The prevalence of malnutrition among patients with gastric cancer ranges from approximately 19–70.6%, influenced by factors like malnutrition risk screening methods, chemotherapy, cancer stages, and age (13–16). Gastric cancer-related malnutrition could result in poorer treatment outcomes (17) and quality of life for patients (18, 19), an increased risk of postoperative complications (12, 20), longer hospital stays (21, 22), and reduced long-term survival rates (23, 24). Therefore, the European Society for Clinical Nutrition and Metabolism (ESPEN) expert group has recommended early malnutrition screening and corresponding individualized nutritional support for all cancers (25). Actually, malnutrition has not received sufficient attention in clinical practice, with 50% of diagnosed cases remaining untreated (26).

**Figure 1:**
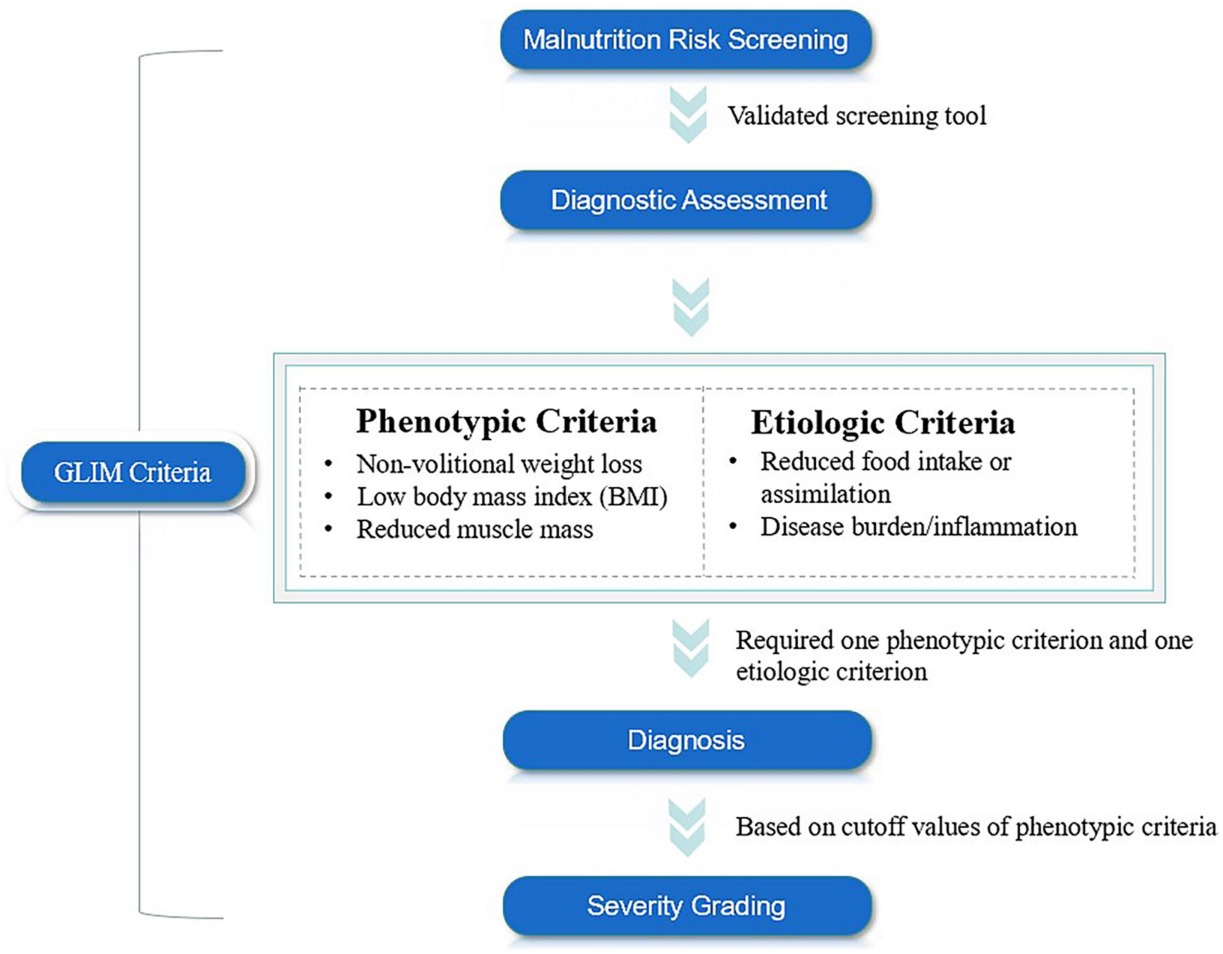
The diagnostic pathway for malnutrition defined by GLIM.

In response to this issue, the Global Leadership Initiative on Malnutrition (GLIM), initiated by multiple nutrition societies and clinical experts, aimed to establish a unified and clinically applicable minimum diagnostic criteria for malnutrition worldwide (27). The GLIM was officially released in 2018 and consists of three main steps: initial screening for nutritional risk using validated tools, such as Nutritional Risk Screening-2002 (NRS-2002), Mini Nutritional Assessment-Short Form (MNA-SF), Malnutrition Universal Screening Tool (MUST). Subsequently, malnutrition can be diagnosed when at least one of the following three phenotypic criteria—non-volitional weight loss, low body mass index (BMI), and muscle mass loss—and at least one of the following two etiological criteria—reduced food intake or absorption, inflammation, or disease burden—are met concurrently. Finally, the severity of malnutrition can be classified as moderate or severe based on the phenotypic criteria (Figure 1) (28). However, this new malnutrition diagnostic tool still faces several challenges, including a lack of consensus on the muscle mass assessment tools and the corresponding cutoff values (29). Additionally, it remains unclear which combination of GLIM diagnostic criteria and nutritional risk screening tools is most appropriate for various populations (30). Furthermore, the actual diagnostic value of this tool across different regions, races, and diseases has yet to be established.

Considering the negative impact of malnutrition on the treatment effect and quality of life of patients with gastric cancer, as well as the current large cohort of East Asian patients with gastric cancer. This review aimed to synthesize existing literature, evaluate the diagnostic validity and prognostic value of the GLIM criteria in East Asian patients with gastric cancer, and further promote the application of this new nutritional status diagnostic tool in Asian populations.

## 2 Materials and methods

### 2.1 Database searching

A comprehensive search was conducted in PubMed, Cochrane Library, and Web of Science for articles related to ‘gastric cancer’ and the ‘Global Leadership Initiative on Malnutrition’ from the inception of each database up to May 31, 2024. All entry terms of gastric cancer were considered in the search. The included studies were published between 2021 and 2024. The detailed literature search methods were listed in Table 1.

**Table 1:** The search strategies of the English database.

#1	“Stomach Neoplasms”[Mesh]
#2	(((((((((((((((((Stomach Neoplasms[Title/Abstract]) OR (Neoplasm, Stomach[Title/Abstract])) OR (Stomach Neoplasm[Title/Abstract])) OR (Gastric Neoplasms[Title/Abstract])) OR (Gastric Neoplasm[Title/Abstract])) OR (Neoplasm, Gastric[Title/Abstract])) OR (Neoplasms, Gastric[Title/Abstract])) OR (Neoplasms, Stomach[Title/Abstract])) OR (Cancer of Stomach[Title/Abstract])) OR (Stomach Cancers[Title/Abstract])) OR (Cancer of the Stomach[Title/Abstract])) OR (Gastric Cancer[Title/Abstract])) OR (Cancer, Gastric[Title/Abstract])) OR (Cancers, Gastric[Title/Abstract])) OR (Gastric Cancers[Title/Abstract])) OR (Stomach Cancer[Title/Abstract])) OR (Cancers, Stomach[Title/Abstract])) OR (Cancer, Stomach[Title/Abstract])
#3	((Global Leadership Initiative on Malnutrition[Title/Abstract]) OR (GLIM[Title/Abstract])) OR (GLIM criteria[Title/Abstract])
#4	#3 AND #2

### 2.2 Eligible criteria

**Figure 2:**
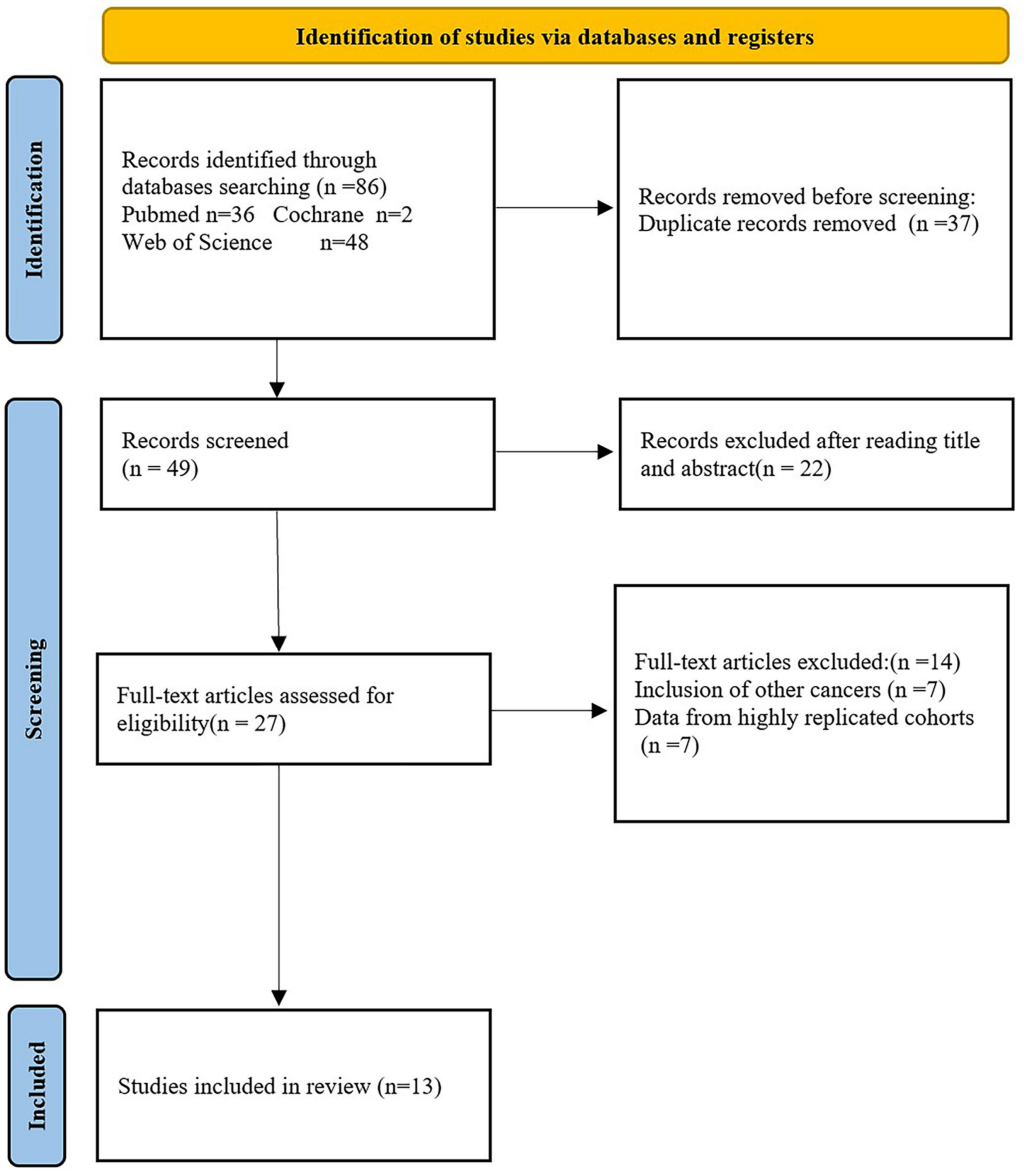
Flow diagram of the study screening process.

(1) The included studies only involved East Asian patients with pathologically diagnosed gastric cancer. (2) Malnutrition diagnosis based on GLIIM criteria. (3) The included studies did not include literature written in languages other than English. (4) Study types included case–control, cross-sectional, and cohort studies. (5) In cases where there was significant overlap in cohort populations, priority was given to studies with larger sample sizes and more standardized and objective results. (6) The focus was on the validity of malnutrition based on the GLIM criteria in the East Asian gastric cancer population and its predictive value for long-term prognosis and postoperative complications. Reviews, meta-analyses, and non-human studies were excluded. The detailed literature screening process was shown in Figure 2.

### 2.3 Data extraction

Two experienced researchers (WJ and CJX) independently extracted relevant data from eligible studies, including authorship, publication year, sample size, age, BMI, muscle mass assessment methods, surgery, screening malnutrition risk tools, prevalence rates, and study outcomes. The studies with disagreements were referred to the third author for resolution.

## 3 Results

### 3.1 Search results and study characteristics

As illustrated in Figure 2, 86 records were identified through searches of Web of Science, PubMed, and Cochrane. After removing 37 duplicate entries, 22 articles were excluded based on screening of titles and abstracts, leaving 27 for full-text review. Fourteen studies were further excluded for involving other cancers and data from highly replicated cohorts. Ultimately, 13 studies with 7,679 cases were included in this review. The general characteristics of the included studies can be found in Tables 2, 3.

**Table 2:** Comparative analysis of the GLIM criteria and PG-SGA for diagnosing malnutrition in East Asian patients with gastric cancer.

Author (Year)	Country	Design	Sample size	Age (mean)	BMI^a^ (mean)	Male (%)	Muscle mass assessment^b^	Malnutrition risk screening tool	Prevalence (malnutrition: %)	Conclusion
Qin (2021)	China	Cross-sectional	217	60	NA	57.1%	^1^BIA: FFMI	NA	GLIM: 65% PG-SGA: 74.2%	Cohen’s kappa: 0.483, moderate agreement
Xu (2021)	China	Prospective	895	64	23.4	76.6%	^2^CT: SMI	MUST	GLIM: 38.3% PG-SGA: 55.2%	Cohen’s kappa: 0.548, moderate agreement
Zheng (2023)	China	Retrospective	1,308	60	NA	71.33%	^3^CC MAC MAMC HGS/W	NA	GLIM: 68.81% PG-SGA: 76.76% Bayesian LCM: 76%	Bayesian LCM (mean sensitivity/specificity) GLIM (0.78/0.64) PG-SGA (0.96/0.87)
Fu (2024)	China	Cross-sectional	405	NA	NA	71.9%	^4^CC BIA: ASMI	NRS-2002	GLIM: 51.9% (CC) GLIM: 53.8% (ASMI) PG-SGA: 70.9%	CC/ASMI: Cohen’s kappa: 0.463/0.470 moderate agreement Specificity: 0.873/0.865 AUC: 0.776/0.781 PPV: 0.929/0.929

FFMI, fat-free mass index; BIA, bioelectrical impedance analysis; SMI, skeletal muscle mass index; BMI, body mass index; CT, computed tomography; Bayesian LCM, Bayesian Latent Class Model; MUST, Malnutrition Universal Screening Tool; NA, not applicable; CC, calf circumference; MAC, mid-arm circumference; MAMC, mid-arm muscle circumference; HGS/W, hand grip strength/weight ratio; NRS-2002, Nutritional Risk Screening-2002; PG-SGA, Patient-Generated Subjective Global Assessment; GLIM, Global Leadership Initiative on Malnutrition; ASMI, appendicular skeletal muscle mass index; PPV, positive predictive value; AUC, area under the curve.

a Low BMI of the Asian standard: (<18.5 if <70 years or <20 if >70 years).

b The definition of reduced muscle mass.

1 FFMI: <15 kg/m^2^ for women, <17 kg/m^2^ for men.

2 SMI: ≤40.8 cm^2^/m^2^ for men and ≤34.9 cm^2^/m^2^.

3 Females: CC < 33.40 cm, MAC < 20.20 cm, MAMC < 19.18 cm, and HGS/W < 0.51; Males: CC < 34.20 cm, MAC < 22.80 cm, MAMC <24.29 cm, and HGS/W < 0.56.

4 CC < 30 cm (male) or <29.5 cm (female), ASMI <7 kg/m^2^ (male) or <5.7 kg/m^2^ (female).

**Table 3:** The predictive value of the GLIM criteria for short- and long-term prognosis in East Asian patients with gastric cancer.

Author (Year)	Country	Design	Sample size	Age (mean)	BMI (mean)	Muscle mass assessment	Radical gastrectomy	Malnutrition risk screening tool	Prevalence (malnutrition: %)	Short and long-term outcomes (multivariate analyses)
Li (2021)	China	Retrospective	877	59.2	NA	CC MAC HGS/W	425	NRS-2002	52.9% (Moderate: 25.9%) (Severe: 27%)	OS: Moderate malnutrition (HR 1.13, 95% CI 0.86–1.47, *p* = 0.372); Severe malnutrition (HR 1.32, 95% CI 1.02–1.71, *p* = 0.038)
Huang (2022)	China	Prospective	1,359	66	22.48	SMI HGS	ALL	NRS-2002	SMI: 28.2% HGS: 27.5% (Severity: NA)	SMI: OS (HR 1.753, 95% CI 1.376–2.232, *p* < 0.001); DFS (HR 1.536, 95% CI 1.215–1.942, *p* < 0.001); Postoperative Complications (OR 1.339, 95% CI 1.012–1.771, *p* = 0.041) HGS: OS (HR 1.766, 95% CI 1.382–2.256, *p* < 0.001); DFS (HR 1.525, 95% CI 1.201–1.937, *p* < 0.001); Postoperative Complications (OR 1.387, 95% CI 1.046–1.840, *p =* 0.023)
Matsui (2022, 2023)	Japan	Retrospective	512	67.93	22.75	SMI	ALL	NA	33.6% (Moderate: 16.4%) (Severe: 17.2%)	OS: Moderate malnutrition (HR 1.689, 95% CI 1.017–2.576, *p* = 0.015); Severe malnutrition (HR 1.918, 95% CI 1.275–2.884, *p* = 0.002) OCS: Moderate malnutrition (HR 2.100, 95% CI 0.904–4.880, *p* = 0.085); Severe malnutrition (HR 3.310, 95% CI 1.426–7.682, *p* = 0.005)
Zhang (2022)	China	Retrospective	182	62	NA	SMI	ALL	NRS-2002	Before NT: 36.3%; After NT: 30.3% (Severity: NA)	Before NT: OS (HR 2.635, 95% CI 1.527–4.527, *p* < 0.001); DFS (HR 2.038, 95% CI 1.252–3.319, *p* = 0.004) After NT: OS (HR 1.736, 95% CI 1.010–2.985, *p* = 0.046); DFS (HR 1.662, 95% CI 1.009–2.736, *p* = 0.046)
Matsui (2023)	Japan	Retrospective	281	65.03	22.95	SMI	ALL	NA	8.2% (Moderate: 3.56%) (Severe: 4.64%)	RFS: Moderate malnutrition (*p* > 0.05); Severe malnutrition (HR 2.393, 95% CI 1.079–5.307, *p* = 0.032)
Zheng (2023)	China	Prospective	1,121	63.3	21.2	SMI	ALL	NA	69.22% (Moderate: 40.23%) (Severe: 28.99%)	OS: Moderate malnutrition (HR 1.224, 95% CI 0.934–1.605, *p* = 0.143); Severe malnutrition (HR 1.768, 95% CI 1.341–2.329, *p* < 0.001)
Zheng (2023)	China	Retrospective	1,308	60	NA	CC MAC MAMC HGS/W	639	NA	68.81% (Severity: NA)	OS (HR 1.56, 95% CI 1.23–1.97, *p* < 0.001)
Song (2024)	Korea	Retrospective	302	60	23.6	SMI	ALL	NA	23.2% (Severity: NA)	RFS (HR 2.016, 95% CI 1.198–3.395, *p* = 0.008)
Sun (2023)	China	Prospective	220	61.79	22.15	SMI	ALL	NRS-2002	30% (Moderate: 14.5%) (Severe: 15.5%)	Postoperative Complications: Moderate malnutrition (OR 15.682, 95% CI 4.481–54.877, *p* < 0.001); Severe malnutrition (OR 20.554, 95% CI 5.771–73.202, *p* < 0.001)

CC, calf circumference; MAC, mid-arm circumference; MAMC, mid-arm muscle circumference; HGS, Hand-grip strength; HGS/W, hand grip strength/weight ratio; OS, Overall Survival; DFS, Disease-Free Survival; OCS, Other-Cause Survival; RFS, relapse-free survival; SMI, skeletal muscle mass index; NA, not applicable; NRS-2002, Nutritional Risk Screening-2002; HR, Hazard Ratio; NT, neoadjuvant treatment; BMI, body mass index.

### 3.2 Comparative analysis of the GLIM criteria and Patient-Generated Subjective Global Assessment (PG-SGA) for diagnosing malnutrition in East Asian patients with gastric cancer

As shown in Table 2, four studies (14, 31–33) from China reported differences in the prevalence of gastric cancer-related malnutrition between the GLIM criteria and the PG-SGA. The PG-SGA was considered the gold standard for evaluating the validity of the GLIM criteria. In a cross-sectional research conducted by Qin et al. (14), it was found that the prevalence of malnutrition according to the GLIM criteria was 65%, while it was higher at 74.2% based on the PG-SGA. The definition of reduced muscle mass was based on a low fat-free mass index (FFMI). The study revealed a moderate agreement between the two standards (Cohen’s kappa = 0.483). Similarly, a retrospective cohort study by Xu et al. reported comparable results (31). In this study, the prevalence of malnutrition based on GLIM and PG-SGA criteria was 38.3 and 55.2%, respectively, with Cohen’s kappa statistic of 0.548. Muscle mass was evaluated using the skeletal muscle index (SMI) as the primary indicator. Zheng et al. also conducted a prospective analysis of GLIM criteria for gastric cancer-related malnutrition, involving 1,308 patients (32). The assessment of muscle mass was based on calf circumference (CC), mid-arm circumference (MAC), mid-arm muscle circumference (MAMC), and hand grip strength/weight ratio (HGS/W). Compared with the GLIM-positive rate of only 68.81%, the PG-SGA has a higher prevalence of 76.76%. Meanwhile, the PG-SGA showed higher sensitivity (0.96) and specificity (0.87) in malnutrition assessment compared with GLIM criteria (0.78/0.64), according to the Bayesian latent class model. In a cross-sectional study by Fu et al. (33), it was found that the GLIM criteria based on NRS-2002 was moderately consistent with the results of PG-SGA, with CC and appendicular skeletal muscle mass index (ASMI) used as the parameter for muscle assessment (Cohen’s kappa for CC/ASMI: 0.463/0.470). The specificity for CC and ASMI was 0.873 and 0.865, while the positive predictive value (PPV) for both measures was 0.929. Additionally, the area under the curve (AUC) for CC and ASMI was 0.776 and 0.781, respectively. The prevalence of malnutrition defined by GLIM was lower than that identified by PG-SGA, with rates of 51.9 and 53.8% for CC and ASMI, compared to 70.9% for PG-SGA. The BMI and weight loss cutoff values in the four studies were based on the criterion recommended by the GLIM guidelines.

### 3.3 The predictive value of the GLIM criteria for long-term outcomes in East Asian patients with gastric cancer

In total, nine studies (29, 32, 34–40) that met the eligibility criteria reported the effects of the GLIM criteria on long-term efficacy in East Asian gastric cancer populations. Among these studies, 5 were conducted in China, 3 in Japan, and 1 in Korea (Table 3). Overall survival (OS) was reported in six, disease-free survival (DFS) and relapse-free survival (RFS) in two studies respectively, and other-cause survival (OCS) in one study.

Six studies focusing on OS demonstrated that GLIM-defined malnutrition (GM) was an independent negative prognostic factor by multivariate analysis (29, 32, 34, 35, 37, 39). There is a consensus among the studies that severe malnutrition was associated with worse OS outcomes (29, 34, 39), despite differences in study type, muscle mass assessment, and malnutrition risk screening tool. However, two large cohort studies showed that moderate malnutrition defined by GLIM did not have a statistically significant effect on OS (34, 39) except for Matsui’s conclusion (29).

A retrospective study conducted by Li revealed that moderate malnutrition did not significantly affect OS, whereas severe malnutrition was linked to a poorer OS (HR 1.32, 95% CI 1.02–1.71, *p* = 0.038) (34). The assessment of muscle mass utilized CC, MAC, and HGS. Similarly, Zheng’s study (39) reported that moderate malnutrition was not associated with OS, while severe malnutrition negatively impacted OS (HR 1.768, 95% CI 1.341–2.329, *p* < 0.001), with SMI used as the parameter for muscle assessment. Additionally, Zheng also found that GM would be a negative prognostic factor for OS, and MAC, MAMC, and HGS/W were used as muscle mass evaluation parameters (32). A retrospective cohort study with 512 Japanese patients by Matsui found an inverse correlation between the severity of malnutrition and OS (29). The definition of muscle mass was based on SMI. Notably, owing to the lack of consensus on the cutoff value of muscle mass, this study graded the severity of malnutrition based solely on BMI and body weight loss (BWL) rate. Interestingly, it was the only study to demonstrate a statistically significant inverse association between moderate malnutrition and OS (HR 1.689, 95% CI 1.017–2.576, *p* = 0.015). Furthermore, severe malnutrition was associated with a poorer prognostic risk (HR 1.918, 95% CI 1.275–2.884, *p* = 0.002). The largest prospective cohort study with 1,359 patients by Huang published in 2022 showed that in addition to SMI-GLIM malnutrition (HR 1.753, 95% CI 1.376–2.232, *p* < 0.001) being associated with OS, GLIM using HGS (HR 1.766, 95% CI 1.382–2.256, *p* < 0.001) has a poor prognostic value for patients undergoing radical gastrectomy (35). Muscle mass was determined based on SMI and HGS. Additionally, GM was also a negative factor associated with reduced DFS (HR for SMI 1.536, 95% CI 1.215–1.942, *p* < 0.001; HR for HGS 1.525, 95% CI 1.201–1.937, *p* < 0.001). Based on the high consistency of the modified criteria in prognostic risk assessment, it is shown that HGS can be used as a simple alternative when evaluating muscle mass is challenging. Zhang conducted a retrospective study showing that before neoadjuvant treatment (NT) and after NT in GM patients would significantly worsen OS and DFS (37), with muscle mass defined using SMI. Furthermore, Matsui found that OCS was also inversely related to severe malnutrition in the previous cohort (HR 3.310, 95% CI 1.426–7.682, *p* = 0.005) (36). Conversely, the impact of moderate malnutrition on OCS was not found to be statistically significant.

As for RFS, Song et al.’s study also supported the negative impact of malnutrition on RFS (HR 2.016, 95% CI 1.198–3.395, *p* = 0.008), with muscle mass defined using the SMI (40). In a single-center cohort study by Matsui involving 182 patients with gastric cancer who received radical gastrectomy and postoperative S-1 adjuvant chemotherapy (a oral fluoropyrimidine-based chemotherapy drug composed of tegafur, gimeracil, and oteracil potassium), severe malnutrition was a negative factor for RFS (HR 2.393, 95% CI 1.079–5.307, *p* = 0.032). In contrast, moderate malnutrition was not statistically significant on RFS (*p* > 0.05) (38).

### 3.4 The predictive value of the GLIM criteria for short-term outcomes in East Asian patients with gastric cancer

As shown in Table 3, two articles (35, 41) examined the predictive value of GM for postoperative complications, utilizing NRS-2002 for nutritional risk screening. Huang et al. found that GM using CT-based SMI or HGS had similar Odds Ratios (1.389; 1.387, respectively) for postoperative complications (Grade II or higher by Clavien-Dindo classification), indicating that GM had an increased risk of postoperative complications (35). A prospective study by Sun et al. focused on severe postoperative complications (SPCs) defined as Grade IIIa or higher by the Clavien–Dindo classification (41). The definition of muscle mass was based on SMI. The analysis revealed that moderate and severe GM were significant risk factors for SPCs, with Odds Ratios of 15.682 and 20.554, respectively. The discrepancies in OR values between the two studies could be attributed to variations in sample sizes and the inclusion of different grades of postoperative complications.

## 4 Limitations of the study

As shown in Tables 2, 3, the diagnostic application of the GLIM criteria varies among included studies, observed in the use of risk screening tools, methods of muscle mass assessment, grading of malnutrition, SMI and CC cutoff values. Although these studies utilized consistent thresholds for non-volitional weight loss and low BMI, differences in the GLIM diagnostic combination were still observed in most studies due to differences in muscle mass assessment tools and their corresponding cutoff values, except for the study by Zhang (37), Song (40), Huang (35), Xu (31), and Sun (41). Zhang and Song utilized SMI as their muscle mass assessment tool, with sex-specific cutoff values of 52.4 cm^2^/m^2^ for men and 38.5 cm^2^/m^2^ for women. Huang, Xu, and Sun adopted different SMI thresholds of <34.9 cm^2^/m^2^ for females and <40.8 cm^2^/m^2^ for males. Moreover, 5 of the 13 studies (33–35, 37, 41) utilized the NRS-2002 for nutritional risk screening, while one study (31) utilized the MUST. The remaining studies did not perform nutritional risk screening. Consequently, there was notable methodological heterogeneity between the studies. Additionally, there were minor differences in the baseline characteristics of the included populations. Except for Li (34) and Zheng (32), the other studies selected patients with gastric cancer who underwent radical gastrectomy. Zhang’s study focused on postoperative patients with gastric cancer who received neoadjuvant therapy (37). Matsui’s study focused on postoperative patients with gastric cancer who underwent radical gastrectomy with S-1 adjuvant chemotherapy (38). No significant differences in age and gender were observed between the studies. According to the results, GM is a poor prognostic factor for both short-term and long-term outcomes in East Asian patients with gastric cancer. However, moderate GM was not found to be a significant risk factor for OS (34), OCS (36), and RFS (38). Notably, Japanese researchers primarily used BMI and body weight loss to assess the degree of malnutrition, showing statistical significance in the OS for cases of moderate malnutrition (29). In short, the varying criteria of GLIM employed to evaluate patients’ nutritional status and the subtle differences in the baseline populations of various study cohorts reduced the reliability of our conclusion.

PG-SGA is a globally recommended method for assessing malnutrition in cancer patients and has demonstrated high sensitivity in identifying malnutrition in patients with gastric cancer (42, 43). Malnutrition based on PG-SGA is also strongly associated with cancer prognosis and adverse events (24, 43). All included studies used a concurrent criterion validity approach recommended by GLIM guidelines to collect data on malnutrition defined by GLIM while completing the PG-SGA as the reference standard (30). The findings indicated a moderate level of consistency between the two assessment tools. Despite using different criteria for muscle loss in the included studies, the diagnosis rate of malnutrition based on the GLIM criteria was lower than the PG-SGA criteria. This discrepancy may be attributed to variations in malnutrition indicators between the two methods and the application of the screening tool before GLIM but not before PG-SGA. For example, a previous study on malnutrition of colorectal cancer patients discovered that utilizing four risk screening tools, including the Malnutrition Screening Tool, PG-SGA short form, NRS-2002, or MUST, led to varying rates of malnutrition defined by GLIM within the identical patient cohort (44). Similar disparities have been observed in other research (45–47). Additionally, the results from various muscle measurement tools for the same patient cohort revealed significant discrepancies in the prevalence of low muscle mass, with 13% identified using mid-upper arm muscle area measurement and 93% of bio-electrical impedance analysis (48). While the diagnostic value of PG-SGA has been confirmed, the validity of GLIM may be skewed compared to comprehensive nutrition assessment. Moreover, the studies also differed in the parameters used to assess the diagnostic performance of the GLIM malnutrition tool. Specifically, the studies by Qin et al. (14) and Xu et al. (31) primarily focused on the kappa statistic, while Zheng et al. (32) emphasized sensitivity and specificity. Fu et al. (33) extended their analysis to include kappa, sensitivity, AUC, and PPV. While these studies demonstrated aspects of the diagnostic performance of the GLIM criteria, including moderate agreement with PG-SGA in some studies based on Kappa values, the parameters commonly used to assess its concurrent validity are still limited. Therefore, future research should aim to perform a more comprehensive evaluation, incorporating sensitivity, specificity, PPV/NPV, ROC curve, accuracy, and positive/negative likelihood ratios, to provide a more complete understanding of GLIM’s diagnostic capabilities. Simultaneously, given the impact of malnutrition on the prognosis of patients with gastric cancer, it is still advisable to implement appropriate nutritional intervention and regular assessment of nutritional status for patients with diagnostic disagreements. Moreover, most current studies are limited by small sample sizes and single-center designs, with some being influenced by the potential bias of retrospective data.

## 5 Future directions for GLIM improvement

Previous studies have investigated the prevalence and prognostic value of GM in gastric cancer populations of different age groups and inflammatory states (49, 50). The results showed that the incidence of malnutrition was higher in the elderly and was not related to inflammatory status. Additionally, advanced age and non-inflammatory status were identified as negative prognostic factors for OS. A propensity score-matched study involving 1,007 postoperative patients with gastric cancer revealed that the long-term outcomes of GM patients with advanced age, female, advanced gastric cancer, and comorbidities were poorer (51). Moreover, a retrospective cohort study showed that colorectal cancer patients with GM and visceral obesity also had the highest risk of long-term prognosis and postoperative complications (52). These findings implied that the prevalence and prognostic implications of GM could vary significantly among patients with gastric cancer with diverse backgrounds, including cancer stage, history of surgery, obesity, edema, presence of metastasis or chronic illnesses. Hence, further investigation is needed to understand the key risk factors associated with poor prognosis in patients with gastric cancer and GM.

There is currently no consensus on the malnutrition risk screening tools for gastric cancer. Furthermore, some included studies did not strictly adhere to the GLIM process for diagnosing malnutrition, which is considered inappropriate. In several studies focusing on nutritional assessment in patients with gastrointestinal tumors, the prevalence of malnutrition diagnosed directly using GLIM was found to be significantly higher than the prevalence of GM based on risk screening (33, 44). Meanwhile, previous research has indicated that GLIM has lower specificity than NRS-2002 in gastrointestinal cancer populations within the same cohort (32, 53), suggesting that neglecting malnutrition screening may lead to an increase in false positive results. In a prospective study evaluating the optimal malnutrition risk screening tool for patients with gastrointestinal tumors, NRS-2002 exhibited exceptionally high specificity and sensitivity among individuals under 65 years (54). Additionally, Wu et al. conducted a study to validate the most suitable risk screening tool for GLIM in patients with colorectal cancer (55). The results showed that the patients with GM diagnosed based on NRS-2002 had the worst impact on long-term prognosis and the best AUC of 0.83. Therefore, it is recommended that future studies adhere to the GLIM process and further validate the value of NRS2002 as a risk screening tool for patients with gastric cancer.

Currently, there is no consensus on the cutoff value for muscle mass loss. Li (34) used the 5th percentile and 15th percentile of the MAC, CC, and HGS/W of the cohort population to diagnose muscle loss, while Matsui (29) estimated separate cutoff values for SMI for men and women based on the median and 25th percentiles for each group. Notably, one of the challenges in applying GLIM in the clinical practice of East Asian populations is the lack of thresholds of SMI to diagnose malnutrition and categorize its severity (56). SMI calculated by computed tomography (CT) is a common and convenient method for evaluating muscle mass inpatients with gastric cancer and is linked to the prognosis of gastric cancer (57, 58). Huang et al. suggested reference values for SMI in Asian populations, with thresholds of <34.9 cm^2^/m^2^ for females and <40.8 cm^2^/m^2^ for males (59). However, these values have not been extensively validated in larger populations. Xu’s (31) and Sun’s (41) study followed the cutoff value recommended by Huang. Furthermore, Zhang (37) and Song (40) used sex-specific cut-off values for SMI of 52.4 cm^2^/m^2^ for men and 38.5 cm^2^/m^2^ for women, which is the most commonly used definition in prognosis studies among non-Asian cancer patients (60). The cut-off values of Matsui’s research as defined by the 25th percentile for SMI were 37.33 cm^2^/m^2^ for males and 29.79 cm^2^/m^2^ for females. Given the lack of cutoff values of SMI for muscle loss for diagnosing GM in the Asian population, further research is needed.

Recent studies have found that the HGS and SMI have shown high consistency in diagnosing malnutrition and assessing prognostic risk, indicating that HGS may become a second-line option for muscle mass assessment (35, 61). Additionally, incorporating gait speed, visceral adipose tissue, and HGS could enhance the predictive value of the GLIM criteria in the prognosis for postoperative patients with gastric cancer (37, 62, 63). Furthermore, previous research on overweight colorectal cancer patients indicated that GLIM had limited diagnostic accuracy for malnutrition due to the influence of high BMI. The addition of low HGS to the GLIM criteria improved the accuracy of diagnosing malnutrition in this population (64). This was also found in Huang’s study that the existing GLIM diagnostic criteria are insufficient to predict the prognostic risk of obese patients (63). These findings indicated that combining GLIM with different measurement indicators can improve the accuracy of malnutrition diagnosis and risk assessment and improve the situation where GLIM is ineffective in some patients. Moreover, the GLIM criteria offer a variety of diagnostic combinations for patients with gastric cancer. Depending on specific diagnostic and treatment requirements, such as focusing on malnutrition assessment or prognostic risk evaluation, the selection of diagnostic combinations may differ. For instance, Li’s study revealed that severe GM diagnosed based on MAC or HGS/W had the poorest long-term prognosis compared to other severe GM diagnostic combinations (34). Furthermore, Brazilian researchers investigated the effects of GM based on various combinations of muscle mass loss on the outcomes in CRC patients (65). The multivariate analysis revealed notable differences in diagnostic efficiency and mortality rates. Consequently, it is of great clinical significance to find out the emphasis of different GM combinations on diagnostic needs to refine the application scenarios of GLIM.

In summary, further large-scale, high-quality, multi-center prospective studies are needed to address these challenges. Meanwhile, establishing a comprehensive database will be essential in improving the GLIM criteria to meet diverse diagnostic requirements.

## 6 Conclusion

In brief, our findings demonstrated that there is insufficient data on sensitivity and specificity to fully validate the GLIM criteria for diagnosing malnutrition in East Asian patients with gastric cancer. Although some studies have reported moderate agreement between the GLIM and the PG-SGA, further research is essential to evaluate the diagnostic validity of GLIM by employing more comprehensive validity parameters, including sensitivity, specificity, and PPV/NPV. Furthermore, GM is associated with poorer short and long-term outcomes. However, due to the lack of consensus on the cutoff value for muscle loss, the impact of moderate malnutrition defined by GLIM on the long-term outcomes of gastric cancer remains controversial. Given the current limitations of GLIM in patients with gastric cancer, high-quality, large-scale, long-term follow-up multicenter prospective studies are needed to verify the predictive value of GM in the East Asian gastric cancer population and to further optimize the GLIM criteria.

## References

[1] Sexton RE Hallak MNA Uddin MH Diab M Azmi AS. Gastric cancer heterogeneity and clinical outcomes. Technol Cancer Res Treat. (2020) 19:1533033820935477. doi: 10.1177/1533033820935477, PMID: 32799763 PMC7432987

[2] Sun DQ Yang F Li H Cao MM Yan XX He SY. Regional disparities in trends of global gastric cancer incidence and mortality from 1990 to 2019. Zhonghua Zhong Liu Za Zhi. (2022) 44:950–4. doi: 10.3760/cma.j.cn112152-20220120-00049, PMID: 36164696

[3] Sung H Ferlay J Siegel RL Laversanne M Soerjomataram I Jemal A. Global cancer statistics 2020: GLOBOCAN estimates of incidence and mortality worldwide for 36 cancers in 185 countries. CA Cancer J Clin. (2021) 71:209–49. doi: 10.3322/caac.21660, PMID: 33538338

[4] Mithany RH Shahid MH Manasseh M Saeed MT Aslam S Mohamed MS. Gastric cancer: a comprehensive literature review. Cureus. (2024) 16:e55902. doi: 10.7759/cureus.55902, PMID: 38595903 PMC11003650

[5] Ilic M Ilic I. Epidemiology of stomach cancer. World J Gastroenterol. (2022) 28:1187–203. doi: 10.3748/wjg.v28.i12.1187, PMID: 35431510 PMC8968487

[6] Yang WJ Zhao HP Yu Y Wang JH Guo L Liu JY. Updates on global epidemiology, risk and prognostic factors of gastric cancer. World J Gastroenterol. (2023) 29:2452–68. doi: 10.3748/wjg.v29.i16.2452, PMID: 37179585 PMC10167900

[7] López MJ Carbajal J Alfaro AL Saravia LG Zanabria D Araujo JM. Characteristics of gastric cancer around the world. Crit Rev Oncol Hematol. (2023) 181:103841. doi: 10.1016/j.critrevonc.2022.103841, PMID: 36240980

[8] Triantafillidis JK Papakontantinou J Antonakis P Konstadoulakis MM Papalois AE. Enteral nutrition in operated-on gastric cancer patients: an update. Nutrients. (2024) 16:1639. doi: 10.3390/nu16111639, PMID: 38892572 PMC11174039

[9] Cederholm T Bosaeus I Barazzoni R Bauer J Van Gossum A Klek S. Diagnostic criteria for malnutrition - an ESPEN consensus statement. Clin Nutr. (2015) 34:335–40. doi: 10.1016/j.clnu.2015.03.001, PMID: 25799486

[10] Cederholm T Barazzoni R Austin P Ballmer P Biolo G Bischoff SC. ESPEN guidelines on definitions and terminology of clinical nutrition. Clin Nutr. (2017) 36:49–64. doi: 10.1016/j.clnu.2016.09.004, PMID: 27642056

[11] Kubota T Shoda K Konishi H Okamoto K Otsuji E. Nutrition update in gastric cancer surgery. Ann Gastroenterol Surg. (2020) 4:360–8. doi: 10.1002/ags3.12351, PMID: 32724879 PMC7382435

[12] Xu R Chen XD Ding Z. Perioperative nutrition management for gastric cancer. Nutrition. (2022) 93:111492. doi: 10.1016/j.nut.2021.111492 34655954

[13] Zhu MW Wei JM Chen W Yang X Cui HY Zhu SN. Dynamic investigation of nutritional risk in patients with malignant tumor during hospitalization. Zhonghua Yi Xue Za Zhi. (2018) 98:1093–8. doi: 10.3760/cma.j.issn.0376-2491.2018.14.009, PMID: 29690722

[14] Qin L Tian Q Zhu W Wu B. The validity of the GLIM criteria for malnutrition in hospitalized patients with gastric cancer. Nutr Cancer. (2021) 73:2732–9. doi: 10.1080/01635581.2020.1856894 33305620

[15] Crestani MS Stefani GP Scott LM Steemburgo T. Accuracy of the GLIM criteria and SGA compared to PG-SGA for the diagnosis of malnutrition and its impact on prolonged hospitalization: a prospective study in patients with cancer. Nutr Cancer. (2023) 75:1177–88. doi: 10.1080/01635581.2023.2184748, PMID: 36892543

[16] Nakyeyune R Ruan X Shen Y Shao Y Niu C Zang Z. Diagnostic performance of SGA, PG-SGA and MUST for malnutrition assessment in adult cancer patients: a systematic literature review and hierarchical Bayesian meta-analysis. Nutr Cancer. (2022) 74:903–15. doi: 10.1080/01635581.2021.1942080, PMID: 34187251

[17] Arends J Bachmann P Baracos V Barthelemy N Bertz H Bozzetti F. ESPEN guidelines on nutrition in cancer patients. Clin Nutr. (2017) 36:11–48. doi: 10.1016/j.clnu.2016.07.015 27637832

[18] Rinninella E Cintoni M Raoul P Pozzo C Strippoli A Bria E. Effects of nutritional interventions on nutritional status in patients with gastric cancer: a systematic review and meta-analysis of randomized controlled trials. Clin Nutr ESPEN. (2020) 38:28–42. doi: 10.1016/j.clnesp.2020.05.007 32690170

[19] Maia FCP Silva TA Generoso SV Correia M. Malnutrition is associated with poor health-related quality of life in surgical patients with gastrointestinal cancer. Nutrition. (2020) 75-76:110769. doi: 10.1016/j.nut.2020.110769, PMID: 32272362

[20] Zhang Y Zhang J Zhu L Hao J He F Xu T. A narrative review of nutritional therapy for gastrointestinal cancer patients underwent surgery. J Investig Surg. (2023) 36:2150337. doi: 10.1080/08941939.2022.2150337, PMID: 36451615

[21] Deftereos I Kiss N Isenring E Carter VM Yeung JM. A systematic review of the effect of preoperative nutrition support on nutritional status and treatment outcomes in upper gastrointestinal cancer resection. Eur J Surg Oncol. (2020) 46:1423–34. doi: 10.1016/j.ejso.2020.04.008, PMID: 32336624

[22] Pimiento JM Evans DC Tyler R Barrocas A Hernandez B Araujo-Torres K. Value of nutrition support therapy in patients with gastrointestinal malignancies: a narrative review and health economic analysis of impact on clinical outcomes in the United States. J Gastrointest Oncol. (2021) 12:864–73. doi: 10.21037/jgo-20-326, PMID: 34012673 PMC8107619

[23] Zhou D Liu Y Zhang L Lu M Gao X Li G. Effects of oral immunonutritional supplement on 3-year disease-free survival in gastric cancer patients with pathological stage III after total gastrectomy (CRUCIAL): study protocol of a multicentre, randomised clinical trial. BMJ Open. (2023) 13:e067990. doi: 10.1136/bmjopen-2022-067990, PMID: 37041057 PMC10106032

[24] Nikniaz Z Somi MH Naghashi S. Malnutrition and weight loss as prognostic factors in the survival of patients with gastric cancer. Nutr Cancer. (2022) 74:3140–5. doi: 10.1080/01635581.2022.2059089, PMID: 35373675

[25] Arends J Baracos V Bertz H Bozzetti F Calder PC Deutz NEP. ESPEN expert group recommendations for action against cancer-related malnutrition. Clin Nutr. (2017) 36:1187–96. doi: 10.1016/j.clnu.2017.06.017 28689670

[26] Caccialanza R Goldwasser F Marschal O Ottery F Schiefke I Tilleul P. Unmet needs in clinical nutrition in oncology: a multinational analysis of real-world evidence. Ther Adv Med Oncol. (2020) 12:1758835919899852. doi: 10.1177/1758835919899852, PMID: 32110247 PMC7025419

[27] Jensen GL Cederholm T Correia M Gonzalez MC Fukushima R Higashiguchi T. GLIM criteria for the diagnosis of malnutrition: a consensus report from the global clinical nutrition community. JPEN J Parenter Enteral Nutr. (2019) 43:32–40. doi: 10.1002/jpen.1440, PMID: 30175461

[28] Cederholm T Jensen GL Correia M Gonzalez MC Fukushima R Higashiguchi T. GLIM criteria for the diagnosis of malnutrition - a consensus report from the global clinical nutrition community. Clin Nutr. (2019) 38:1–9. doi: 10.1016/j.clnu.2018.08.002, PMID: 30181091

[29] Matsui R Inaki N Tsuji T. Impact of GLIM defined malnutrition on long term prognosis in patients with gastric cancer after gastrectomy. Anticancer Res. (2022) 42:4611–8. doi: 10.21873/anticanres.15965, PMID: 36039451

[30] de van der Schueren M Keller H GLIM Consortium Cederholm T Barazzoni R Compher C. Global leadership initiative on malnutrition (GLIM): guidance on validation of the operational criteria for the diagnosis of protein-energy malnutrition in adults. Clin Nutr. (2020) 39:2872–80. doi: 10.1016/j.clnu.2019.12.022, PMID: 32563597

[31] Xu LB Shi MM Huang ZX Zhang WT Zhang HH Shen X. Impact of malnutrition diagnosed using global leadership initiative on malnutrition criteria on clinical outcomes of patients with gastric cancer. JPEN J Parenter Enteral Nutr. (2022) 46:385–94. doi: 10.1002/jpen.2127, PMID: 33908649

[32] Zheng X Ruan X Wang X Zhang X Zang Z Wang Y. Bayesian diagnostic test evaluation and true prevalence estimation of malnutrition in gastric cancer patients. Clin Nutr ESPEN. (2024) 59:436–43. doi: 10.1016/j.clnesp.2023.12.019, PMID: 38220406

[33] Fu L Xu X Zhang Y Jin J Zhu S Shi H. Agreements between GLIM using left calf circumference as criterion for reduced muscle mass and PG-SGA, and GLIM using ASMI for the diagnosis of malnutrition in gastric cancer patients. Nutr Hosp. (2024) 10.20960/nh.05024 38666329

[34] Li Q Zhang X Tang M Song M Zhang Q Zhang K. Different muscle mass indices of the global leadership initiative on malnutrition in diagnosing malnutrition and predicting survival of patients with gastric cancer. Nutrition. (2021) 89:111286. doi: 10.1016/j.nut.2021.111286 34090215

[35] Huang DD Yu DY Wang WB Song HN Luo X Wu GF. Global leadership initiative in malnutrition (GLIM) criteria using hand-grip strength adequately predicts postoperative complications and long-term survival in patients underwent radical gastrectomy for gastric cancer. Eur J Clin Nutr. (2022) 76:1323–31. doi: 10.1038/s41430-022-01109-2, PMID: 35314767

[36] Matsui R Inaki N Tsuji T. Impact of preoperative nutritional assessment on other-cause survival after gastrectomy in patients with gastric cancer. Nutrients. (2023) 15:3182. doi: 10.3390/nu15143182, PMID: 37513603 PMC10386384

[37] Zhang Y Jiang L Su P Yu T Ma Z Kang W. Visceral adipose tissue assessment enhances the prognostic value of GLIM criteria in patients with gastric cancer undergoing radical gastrectomy after neoadjuvant treatment. Nutrients. (2022) 14:5047. doi: 10.3390/nu14235047, PMID: 36501076 PMC9740239

[38] Matsui R Inaki N Tsuji T. Effect of malnutrition as defined by the global leadership initiative on malnutrition criteria on compliance of adjuvant chemotherapy and relapse-free survival for advanced gastric cancer. Nutrition. (2023) 109:111958. doi: 10.1016/j.nut.2022.111958, PMID: 36716614

[39] Zheng HL Lin J Shen LL Yang HB Xu BB Xue Z. The GLIM criteria as an effective tool for survival prediction in gastric cancer patients. Eur J Surg Oncol. (2023) 49:964–73. doi: 10.1016/j.ejso.2023.01.009, PMID: 36958948

[40] Song GJ Ahn H Son MW Yun JH Lee MS Lee SM. Adipose tissue quantification improves the prognostic value of GLIM criteria in advanced gastric cancer patients. Nutrients. (2024) 16:728. doi: 10.3390/nu16050728, PMID: 38474856 PMC10934376

[41] Sun S Huang W Wang Z Xie W Zhou J He Q. Association of malnutrition diagnosed using global leadership initiative on malnutrition criteria with severe postoperative complications after gastrectomy in patients with gastric cancer. J Laparoendosc Adv Surg Tech A. (2023) 33:1193–200. doi: 10.1089/lap.2023.0310, PMID: 37787912

[42] Chen X Liu X Ji W Zhao Y He Y Liu Y. The PG-SGA outperforms the NRS 2002 for nutritional risk screening in cancer patients: a retrospective study from China. Front Nutr. (2023) 10:1272420. doi: 10.3389/fnut.2023.1272420, PMID: 38075213 PMC10702952

[43] Guo ZQ Yu JM Li W Fu ZM Lin Y Shi YY. Survey and analysis of the nutritional status in hospitalized patients with malignant gastric tumors and its influence on the quality of life. Support Care Cancer. (2020) 28:373–80. doi: 10.1007/s00520-019-04803-3, PMID: 31049672 PMC6882767

[44] Henriksen C Paur I Pedersen A Kværner AS Ræder H Henriksen HB. Agreement between GLIM and PG-SGA for diagnosis of malnutrition depends on the screening tool used in GLIM. Clin Nutr. (2022) 41:329–36. doi: 10.1016/j.clnu.2021.12.024, PMID: 34999327

[45] Zhang Z Wan Z Zhu Y Zhang L Zhang L Wan H. Prevalence of malnutrition comparing NRS2002, MUST, and PG-SGA with the GLIM criteria in adults with cancer: a multi-center study. Nutrition. (2021) 83:111072. doi: 10.1016/j.nut.2020.111072 33360034

[46] De Groot LM Lee G Ackerie A van der Meij BS. Malnutrition screening and assessment in the cancer care ambulatory setting: mortality predictability and validity of the patient-generated subjective global assessment short form (PG-SGA SF) and the GLIM criteria. Nutrients. (2020) 12:2287. doi: 10.3390/nu12082287, PMID: 32751724 PMC7468976

[47] Rosnes KS Henriksen C Høidalen A Paur I. Agreement between the GLIM criteria and PG-SGA in a mixed patient population at a nutrition outpatient clinic. Clin Nutr. (2021) 40:5030–7. doi: 10.1016/j.clnu.2021.07.019, PMID: 34365037

[48] Blauwhoff-Buskermolen S Langius JAE Becker A Verheul HMW de van der Schueren MAE. The influence of different muscle mass measurements on the diagnosis of cancer cachexia. J Cachexia Sarcopenia Muscle. (2017) 8:615–22. doi: 10.1002/jcsm.12200, PMID: 28447434 PMC5566652

[49] Matsui R Inaki N Tsuji T Fukunaga T. Association of GLIM defined malnutrition according to preoperative chronic inflammation with long-term prognosis after gastrectomy in patients with advanced gastric cancer. J Clin Med. (2023) 12:1579. doi: 10.3390/jcm12041579, PMID: 36836114 PMC9966663

[50] Matsui R Inaki N Tsuji T. Impact of malnutrition as defined by the global leadership initiative on malnutrition criteria on the long-term prognosis in older patients with gastric cancer after gastrectomy. Surg Today. (2023) 53:578–87. doi: 10.1007/s00595-022-02594-5, PMID: 36131158

[51] Cai W Yang H Zheng J Huang J Ji W Lu Y. Global leaders malnutrition initiative-defined malnutrition affects long-term survival of different subgroups of patients with gastric cancer: a propensity score-matched analysis. Front Nutr. (2022) 9:995295. doi: 10.3389/fnut.2022.995295, PMID: 36245538 PMC9562265

[52] Zhou CJ Lin Y Liu JY Wang ZL Chen XY Zheng CG. Malnutrition and visceral obesity predicted adverse short-term and long-term outcomes in patients undergoing proctectomy for rectal cancer. BMC Cancer. (2023) 23:576. doi: 10.1186/s12885-023-11083-y, PMID: 37349711 PMC10286325

[53] Ruan X Wang X Zhang Q Nakyeyune R Shao Y Shen Y. The performance of three nutritional tools varied in colorectal cancer patients: a retrospective analysis. J Clin Epidemiol. (2022) 149:12–22. doi: 10.1016/j.jclinepi.2022.04.026, PMID: 35537604

[54] Huang Y Chen Y Wei L Hu Y Huang L. Comparison of three malnutrition risk screening tools in identifying malnutrition according to global leadership initiative on malnutrition criteria in gastrointestinal cancer. Front Nutr. (2022) 9:959038. doi: 10.3389/fnut.2022.959038 35990353 PMC9386177

[55] Wu T Xu H Li W Zhou F Guo Z Wang K. The potential of machine learning models to identify malnutrition diagnosed by GLIM combined with NRS-2002 in colorectal cancer patients without weight loss information. Clin Nutr. (2024) 43:1151–61. doi: 10.1016/j.clnu.2024.04.001, PMID: 38603972

[56] Compher C Cederholm T Correia M Gonzalez MC Higashiguch T Shi HP. Guidance for assessment of the muscle mass phenotypic criterion for the global leadership initiative on malnutrition diagnosis of malnutrition. JPEN J Parenter Enteral Nutr. (2022) 46:1232–42. doi: 10.1002/jpen.2366, PMID: 35437785

[57] Rinninella E Cintoni M Raoul P Pozzo C Strippoli A Bria E. Muscle mass, assessed at diagnosis by L3-CT scan as a prognostic marker of clinical outcomes in patients with gastric cancer: a systematic review and meta-analysis. Clin Nutr. (2020) 39:2045–54. doi: 10.1016/j.clnu.2019.10.021, PMID: 31718876

[58] Sánchez-Torralvo FJ Ruiz-García I Contreras-Bolívar V González-Almendros I Ruiz-Vico M Abuín-Fernández J. CT-determined sarcopenia in GLIM-defined malnutrition and prediction of 6-month mortality in cancer inpatients. Nutrients. (2021) 13:2647. doi: 10.3390/nu13082647, PMID: 34444806 PMC8398807

[59] Huang DD Chen XX Chen XY Wang SL Shen X Chen XL. Sarcopenia predicts 1-year mortality in elderly patients undergoing curative gastrectomy for gastric cancer: a prospective study. J Cancer Res Clin Oncol. (2016) 142:2347–56. doi: 10.1007/s00432-016-2230-4, PMID: 27573385 PMC11819390

[60] Prado CM Lieffers JR McCargar LJ Reiman T Sawyer MB Martin L. Prevalence and clinical implications of sarcopenic obesity in patients with solid tumours of the respiratory and gastrointestinal tracts: a population-based study. Lancet Oncol. (2008) 9:629–35. doi: 10.1016/S1470-2045(08)70153-0 18539529

[61] Zhou LP Yu DY Ma BW Shen ZL Zou HB Zhang XZ. Feasibility of substituting handgrip strength for muscle mass as a constituent standard in the global leadership initiative on malnutrition for diagnosing malnutrition in patients with gastrointestinal cancers. Nutrition. (2021) 84:111044. doi: 10.1016/j.nut.2020.111044, PMID: 33517155

[62] Li ZZ Yan XL Zhang Z Chen JL Li JY Bao JX. Prognostic value of GLIM-defined malnutrition in combination with hand-grip strength or gait speed for the prediction of postoperative outcomes in gastric cancer patients with cachexia. BMC Cancer. (2024) 24:253. doi: 10.1186/s12885-024-11880-z, PMID: 38395798 PMC10885679

[63] Huang DD Wu GF Luo X Song HN Wang WB Liu NX. Value of muscle quality, strength and gait speed in supporting the predictive power of GLIM-defined malnutrition for postoperative outcomes in overweight patients with gastric cancer. Clin Nutr. (2021) 40:4201–8. doi: 10.1016/j.clnu.2021.01.038 33583658

[64] Chen W Yu D Ren Q Shen Z Huang G Chen X. Predictive value of global leadership initiative on malnutrition criteria combined with handgrip strength for postoperative outcomes in overweight colorectal cancer patients. J Gastroenterol Hepatol. (2024) 39:716–24. doi: 10.1111/jgh.16481, PMID: 38212102

[65] Dos Santos ALS Santos BC Frazão LN Miranda AL Fayh APT Silva FM. Validity of the GLIM criteria for the diagnosis of malnutrition in patients with colorectal cancer: a multicenter study on the diagnostic performance of different indicators of reduced muscle mass and disease severity. Nutrition. (2024) 119:112324. doi: 10.1016/j.nut.2023.112324, PMID: 38215671

