# Supplementary figures and images for: Validity of the Global Leadership Initiative on Malnutrition criteria in East Asian patients with gastric cancer: a comprehensive narrative review
Source: Front Nutr. 2024 Nov 20;11:1462487. doi: 10.3389/fnut.2024.1462487 (PMC11614637; doi:10.3389/fnut.2024.1462487)

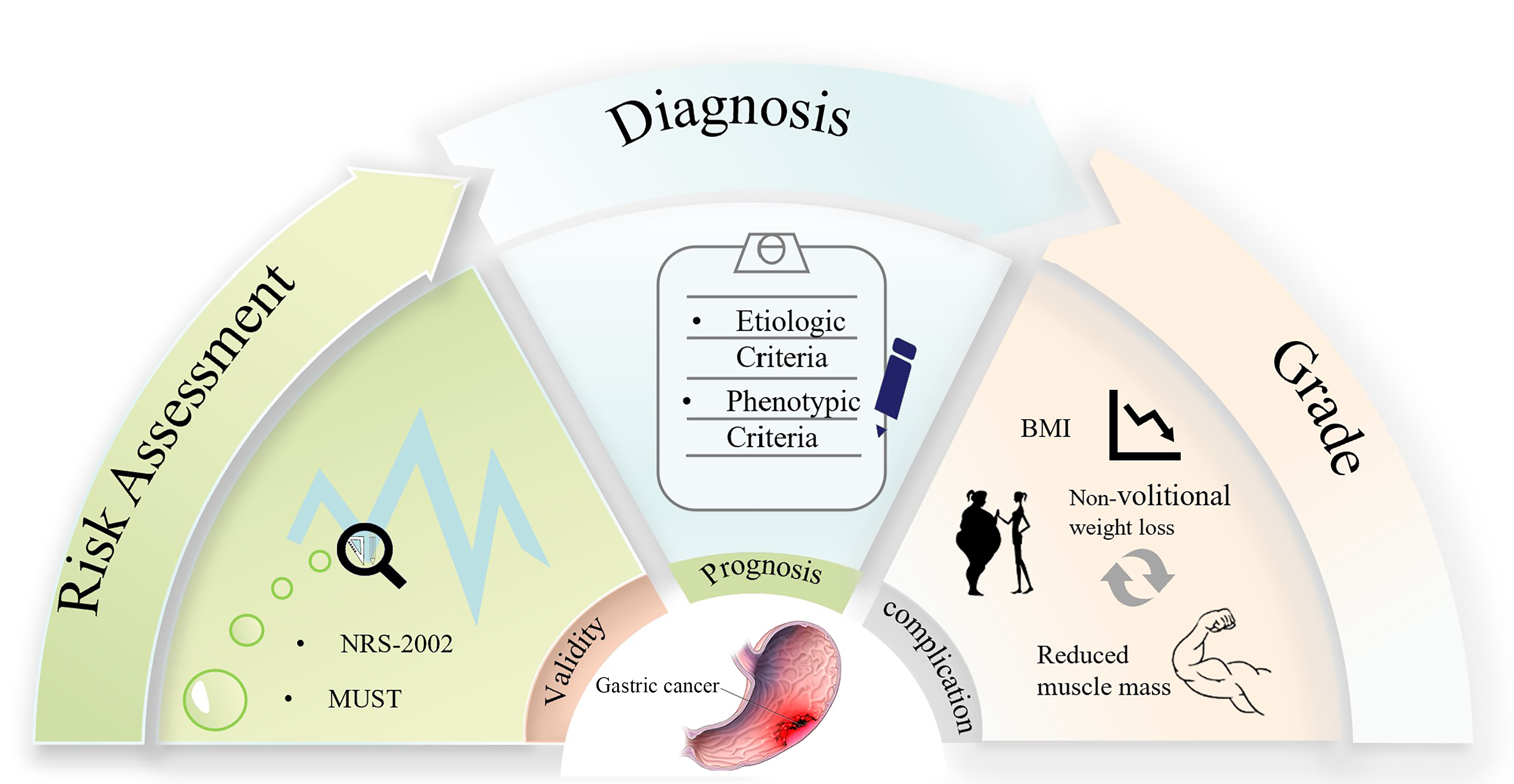

Supplement: Supplementary file 1 [file Image_1.TIF]
